# Estrus detection in dairy cattle: an updated review on strategies and technologies

**DOI:** 10.3389/fvets.2026.1807199

**Published:** 2026-05-27

**Authors:** Devaraj Sankarganesh, Parcha Durga Sai Sita Yesaswini Krishna Prasad, Shivani Sujit, Annis Inncia Gnana Thiraviam, Divakar Justus Ambrose

**Affiliations:** 1 School of Bio Sciences and Technology, Vellore Institute of Technology, Vellore, Tamil Nadu, India; 2 Department of Agricultural, Food and Nutritional Science, University of Alberta, Edmonton, AB, Canada

**Keywords:** estrogen, female dairy cattle, progesterone, silent heat, wearable electronic devices

## Abstract

Many factors may negatively affect dairy cattle productivity, among which imprecise estrus detection is a major contributor to low reproductive efficiency. Given that artificial insemination is widely used in the dairy industry, accurate estrus detection is of great importance. Estrus synchronization using hormonal treatments is a common approach to regulate the estrous cycle and to improve the efficiency of estrus detection. Estrus detection using artificial intelligence-aided data analyses is a new development. Biomarker-based chemical sensors and activity based wearable and implantable sensors are also available for monitoring. Behavioral datasets have been used in machine learning and other approaches; however, such datasets must be validated for their applicability in detecting estrus before being utilized by dairy farmers. The cumulative knowledge of estrus detection approaches, their merits, demerits, applicability, and cost-effectiveness is highly warranted. Therefore, here, we provide an update on the recent approaches and technologies used for estrus detection, particularly in dairy cattle, and discuss emerging experimental technologies with potential for future applications.

## Introduction

Cattle production is important for strengthening the economy and ensuring an appropriate supply of livestock products (1) for the growing human population. Reproductive efficiency is a crucial factor that directly affects dairy cattle production. In addition, detection of estrus in multiparous dairy cattle is challenging due to reduced behavioral signs as compared to primiparous cows (2), and postpartum disease also affects estrus detection (3). Of note, high-milk-yielding Holstein cows had less prominent signs of estrus (4), indicating the negative influence of milk production on estrus expression. Xu et al. (5) observed that autumn-calving cows had a longer estrus period than spring-calving cows, implying that the calving season also affects estrus expression. Housing conditions also regulate the expression of estrus behavior, as evidenced by the fact that tie-stalled cows do not show typical estrus behaviors (6).

In general, estrus is outwardly expressed by specific behaviors identified by male conspecifics (7). However, “silent estrus” is an important issue, occurring at a high rate in dairy cows, which contributes to estrus going undetected by humans (8). Visual observation of estrus signs is a widely adopted method for estrus detection in dairy cattle, but it is labor-intensive and unreliable under silent heat conditions (9). There are also many other factors, such as the environment, herd size, and housing, that can impact estrus detection efficiency (10). Although the use of teaser bulls helps in detecting estrus, their use is limited. Moreover, the use of bulls on large farms is time-consuming and expensive. One study (11) compared the cost of natural service to that of timed artificial insemination (TAI) and found that the net cost (in US Dollars) of using bulls for natural service was $100.49/cow per year versus $67.80/cow per year for TAI. Use of teaser bulls also raises safety concerns for workers (12, 13). Put together, many large-scale farms rely minimally on natural mating systems and adopt artificial insemination (AI) approaches after estrus detection; therefore, estrus detection methods should be robust and efficient to achieve high reproductive efficiency and productivity in these farms.

**Figure 1:**
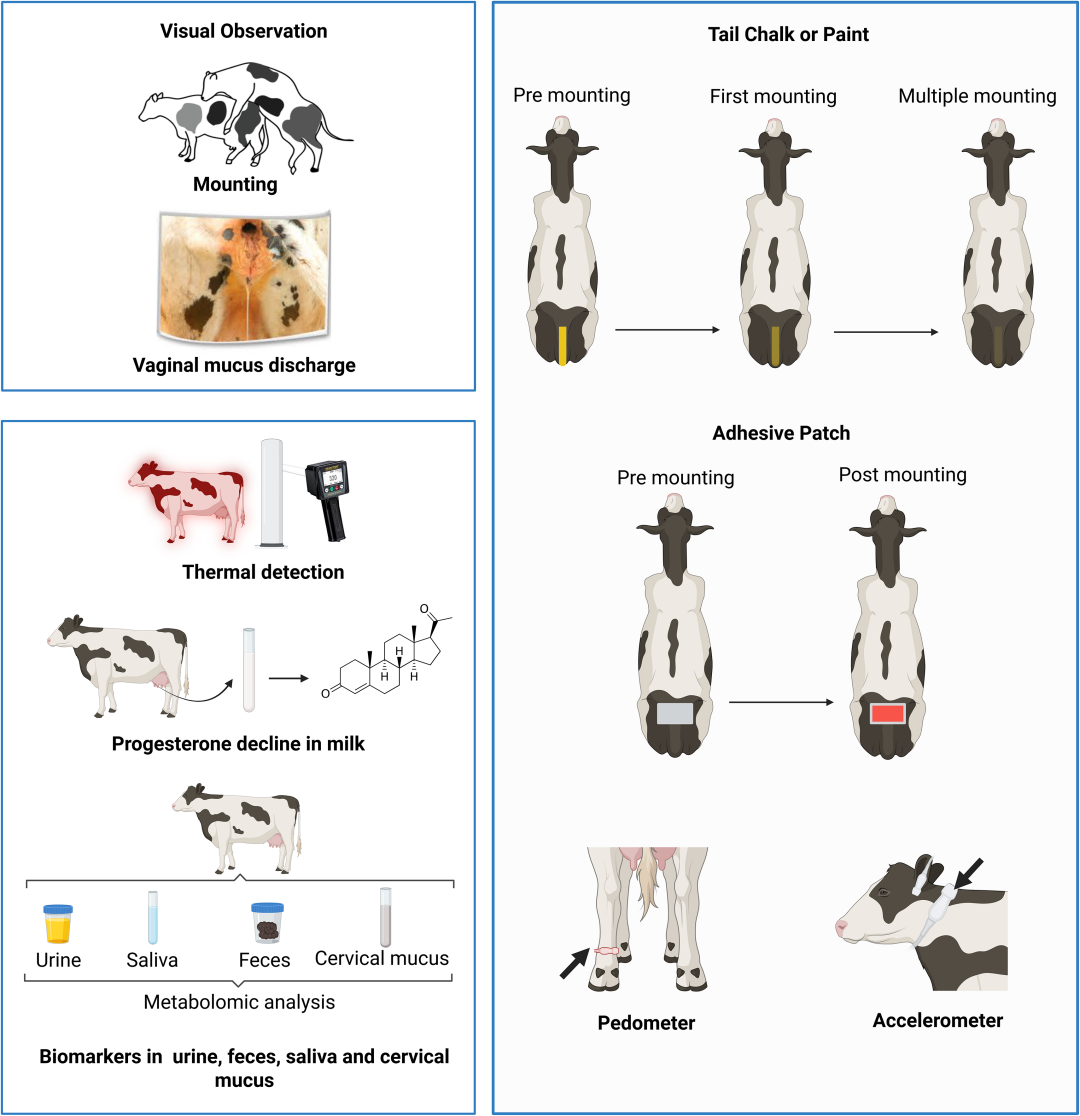
Visual observation based and other estrus detection methods for dairy cows.

**Figure 2:**
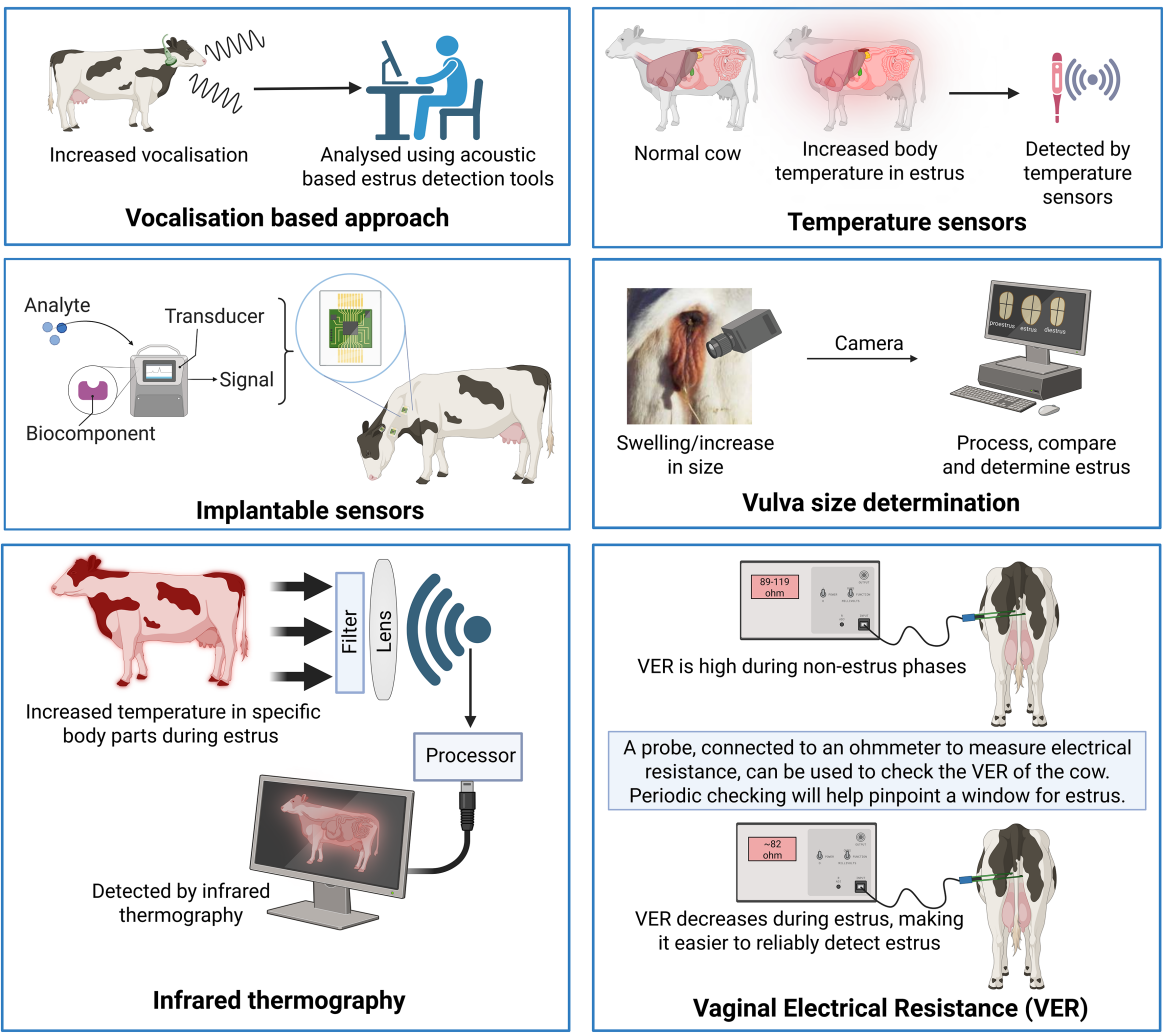
Experimental approaches used in estrus detection of dairy cattle.

Thus, there is considerable demand for the development of practical and cost-effective methods for estrus detection in dairy cattle. Automated estrus detection methods reduce reproductive inefficiencies and economic losses in dairy herds. A recent review (14) reported that precision livestock farming technologies reduce extended calving and insemination intervals, thereby reducing the costs and milk production losses. This important field of research requires interdisciplinary and transdisciplinary approaches. Furthermore, comprehensive reporting is necessary to ascertain the merits and demerits of the available techniques. The documented estrus detection approaches (behavioral, physiological and activity-based indicators) are depicted in Figures 1 and 2. In addition, the advantages and disadvantages of each of the estrus-specific behavior(s) are listed in Table 1. Despite the continuous advancements of various estrus detection methods, the gold standard method is yet to be established.

**Table 1:** Various methods of estrus detection and their advantages and potential disadvantages as inferred from literature.

Indicators	Advantages	Disadvantages	Best use case (small farms, large farms, or experimental use)
Behavioral indicators Visual observation (real-time monitoring) of standing estrus behavior.	Highly practiced and easy to adopt	Observer bias; time-consuming; labor-intensive	Best suitable for small farms
Recorded behaviors (Mounting and Riding) Post-screening Validation by trained technicians/veterinarians Image processing of recorded behaviors	Manual screening of large datasets Faster analysis of data	Observer effect; time consuming; high incidence of false positive; limitations to use in open-housing or grazing system.	Best suitable for small farms
Physiological indicators Vulval mucus congestion Uterine tone and vaginal mucus discharge	Highly used	Depends on parity and other related factors	Best suitable for small farms
Vaginal temperature	Validated technology	Not practical in large farms; may be expensive; time and labor intensive	Best suitable for small farms and experimental use
Electrical conductivity		Requires some training and sensitive equipment; greater risk of spreading disease	Best suitable for small farms and experimental use
Activity based approaches			
Tail paint/ Tail chalk	Inexpensive and highly used	Manual application; Time and labor-intensive; Not best suited for wet weather; Not ideal for large farms	Best suitable for small to medium farms
Estrus detection patches	Proven technology; reasonable cost; weather resistant	Inconsistency in patch retention time	Suitable for short-term application in both small and large farms
Physically fitted sensors. (Accelerometers, mount detectors, pedometers, etc.)	Many varieties available; highly adopted; may detect some cases of “silent estrus”	High cost; Detection depends on the expression of cow behaviors often affected by housing; depends on parity and season; inefficient in tie-stalls	Suitable for both small and large farms
Monitoring real-time milk progesterone concentration using fully automated system	High precision technology; Not widely prevalent	High initial investment and recurring annual costs; limited to robotic milking systems of a specific brand.	Best suitable for small and medium farms
Vocalization-based approaches	Could be highly effective when used in combination with other approaches in small herds	Differentiating between social calls and estrus calls may be difficult. Differentiation of calls in group housed animals could pose problems	Best suitable for small farms
Infrared thermography	Potential for fully automated use	Currently experimental use only; increased false positive rate.	Maybe suitable for both small and large farms
Implantable sensors	Less utilized	Invasive, require surgery, not feasible in large farms	Best suitable for small farms
Chemical signals in body fluids	Field-level applications are limited	Interfering substances in body fluids affect the functioning of the sensor; calibration of the sensor	Could be suitable for small and large farms if the technology becomes adoptable
Machine learning approaches/ algorithms	High precision; ideal for use in small and large farms alike	Early stages of development; require large dataset to be used in the training; relies on the behavioral or physiological indicators; could be expensive initially	Suitable for both small and large farms

## Physiology of estrus and estrus behaviors

### Primary and secondary signs of estrus in dairy cattle

Estrus behaviors are typically categorized into two types: primary and secondary behaviors. In cattle, the primary estrus behavior is standing estrus (standing to be mounted) (15), whereas secondary behaviors are exhibited before, and in some cases, after the onset of standing heat. The expression of secondary estrus behaviors is reduced in cows that do not exhibit primary estrus behavior (16). The expression of primary estrus behavior is dependent on the endocrine status and preceding secondary estrus behaviors (17). Therefore, the complexity of estrus detection is apparent in cows that exhibit weak primary estrus behaviors.

Secondary estrus behaviors help detect estrus in cows that do not present standing estrus. For instance, in Holstein cows, the expression of standing to be mounted was very low; however, the expression of secondary estrus behaviors, such as mounting, chin-resting, and sniffing the anogenital regions, was higher, aiding in estrus detection (18, 19). Sveberg et al. (20) also highlighted sniffing the vulva, chin resting, and mounting and attempting-to-mount as secondary estrus behaviors of Holstein-Friesian cows. Similar to primary estrus behaviors, the expression patterns of secondary behaviors vary depending on various factors. For instance, secondary estrus signs, such as repeated mounting, vaginal mucus discharge, and uterine tonus, were categorized as weak, normal, and strong, respectively, and used to detect estrus (21). One such study reviewed the importance of behavioral parameters in estrus detection and concluded that secondary signs of estrus were more predictive than standing estrus, attesting that secondary signs also complement estrus detection. Secondary signs such as sexual and social behaviors, decreased rumination time, increased activity, and restlessness were used to detect estrus (22). Nevertheless, it is also pertinent to consider that behavior often changes based on various factors (animal- and environment-related, as mentioned elsewhere in this manuscript).

Interestingly, visual observation of recorded primary signs of estrus, at least two secondary signs of estrus, or standing behavior were used to confirm estrus adopting a reference test for behavioral estrus (RTE) (23), implying the use of both primary and secondary behaviors in estrus detection. Studies have also suggested other secondary behaviors, such as rumination/feeding time, lying time, and activity; however, an understanding of the hormonal influence on these behaviors is highly warranted (24). The physiological signs of estrus were highly correlated with the endocrine profile (25). For instance, vocalization has been proposed as a good indicator for estrus detection. It was found that vocalization peaks before the estrus, suggesting a close tie with reproductive receptivity. Higher vocalization rates also correspond to increased estrogen levels, which drive the attraction of males (26). Cervical mucus discharge and the physical appearance of the mucus also serve as signs of estrus in dairy cows but vary between spontaneous and induced estrous cycles. For instance, the viscosity of cervical mucus is decreased, whereas, Spinnbarkeit (elasticity), mucus crystallization (ferning) pattern and sperm penetration are increased in spontaneous estrus than in induced estrus (27). Table 2 summarizes the studies that used a combination of methods to determine the best method for estrus detection.

**Table 2:** Analysis of studies that used combination of methods to derive the best method for estrus detection.

Comparative approaches used	Number of animals	Best method proposed	Reference
Visual observation, tail paint and radiotelemetry	46	All three have the same efficiency	(113)
Visual observation and pressure sensitive detection device	164	Visual observation	(114)
Pedometer and mount detection device	98	Combination of both	(115)
Pedometer and activity meters	63	Pedometer (high sensitivity) Estrus monitoring system (high positive predictive value)	(116)
Mounting detection devices vs. automated estrus detection	1,019	Mounting detection devices	(117)
Visual observation vs. automated mounting monitoring system	421	No difference observed	(23)
Vaginal temperature vs. pedometer	10	Vaginal temperature	(75)
Visual observation and radiotelemetric devices	50	Radiotelemetric devices	(118)
Visual observation, activity sensor and HeatWatch^#^ Device	255	Estrus monitoring system	(119)
Estrus monitoring system, electronic activity tag, pedometer and visual observation	23	Estrus monitoring system, electronic activity tag, pedometer	(120)
Camera-icon method and visual observation	35	Camera-icon method	(86)
Visual observation vs. estrus behavior scoring system (multiple technologies)	109	Estrus behavior scoring system (multiple technologies)	(121)
Visual estrus detection vs. automated estrus detection	139	Automated estrus detection	(7)
Estrus monitoring systems (Heatime and IceTag)	57	Equally efficient	(122)
Visual observation vs. DEC electronic device^^^	30	No difference observed	(123)

The above table evidenced that many studies have utilized various methods to compare their efficiency in estrus detection. The best method was derived based on the observations and results of individual study setup, which normally had many variables (number of cows, parity, age, sex and other variables, season, etc.).

# HeatWatch - DDx Inc., Denver, CO.

^^^ IMV Technologies, France.

### Endocrinological and physiological influence on estrus behaviors

**Figure 3:**
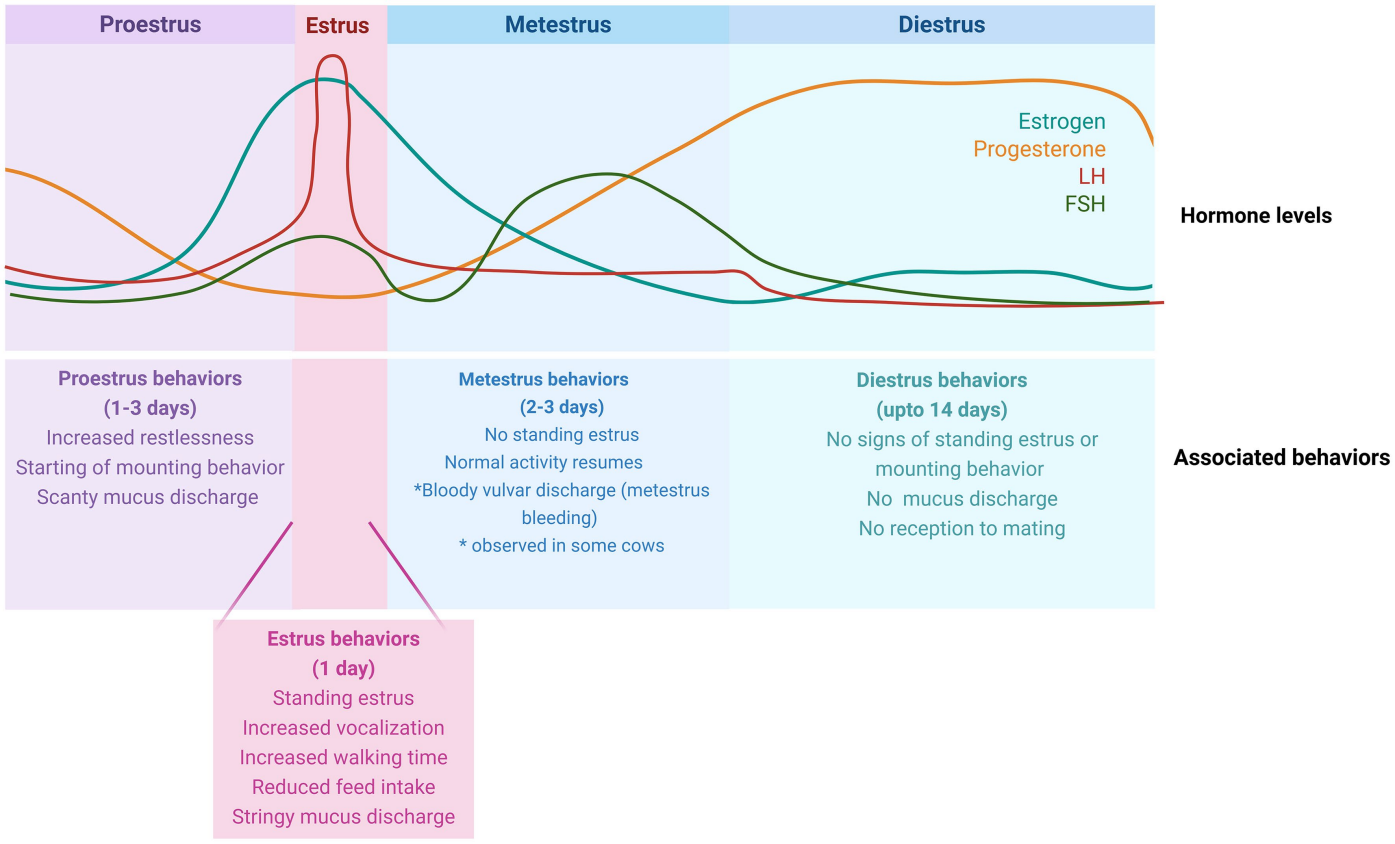
Depicts the stages (pro-estrus, estrus, metestrus and diestrus) of the bovine estrous cycle and their associated behaviors.

The estrous cycle of dairy cows is tightly regulated by their endocrine and physiological statuses. In particular, the endocrine system influences the expression of all behaviors and plays an important role in estrus detection as sexual behaviors (primary and secondary estrus behaviors) and physiological signs (vulval swelling, frequent urination, etc.) are regulated by the endocrine milieu. For instance, the elevated estrogen levels ultimately regulate the activity of cows during estrus (28). Progesterone concentration also influences estrus intensity, wherein high progesterone levels 4 days before estrus and low progesterone levels at estrus (day 0) influence the intensity of estrus expression (29). Therefore, it is understood that the endocrine milieu heavily influences the physiology of dairy cows by influencing the expression of sexual behaviors, including primary and secondary estrus behaviors, and other physiological signs (Figure 3).

## Techniques for estrus detection

### Behavior-based methods

#### Visual observations

Visual observation of estrus behavior has been suggested as a common method for detecting estrus in cows. For instance, mounting by a bull and standing to be mounted by a bull or a female herdmate is noteworthy. In zebu cattle, mucus discharge, vulval swelling, and reddening of the vulval mucous membrane were found to be strong indicators of estrus, as these indicators lasted for a longer duration than mounting behaviors (30). Nevertheless, no automated devices are available to measure these characteristics in dairy cattle. Trained technicians can use uterine tonicity and vaginal mucus discharge as aids in detecting estrus (31); however, these methods are impractical for large herds.

A recent evaluation (32) comparing the traditional visual observation, (TVO), computer vision technique (CV) and activity monitors (ACT), in group-housed Holstein-Friesian cows. They found the precision values as 100, 97.5 and 87.5% for TVO, CV and ACT, respectively. However, the false negatives were high in TVO, implying that visual observations, however accurate they may be, systematically underestimate the estrus prevalence in practical farm settings. Additionally, Mörig et al. (33) reported in a meta-analysis that automated estrus alerts from activity monitoring systems detected over 83% of cows in estrus by 80 days in milk, outperforming visual observation alone, reinforcing the case for supplementing, and eventually replacing manual observation with automated approaches.

#### Tail chalk or paint

Tail chalk and tail paint are widely used methods to detect estrus, in that the partial and complete removal of chalk or paint imply the incidence and intensity of mounting by herdmates. For instance, if the chalk or paint is removed partially, it could indicate that the mounting activity was limited, whereas its complete removal indicates a high frequency of mounting. Similarly, Mendonca et al. (34) used tail painting as an indicator of estrus detection. The limitation of tail chalk or paint, however, is that social behaviors such as playful mounting and licking can partially or fully remove the coloring agent (35). There is also a high possibility that the cows in the open lots and materials used in enrichment of farms (e.g., automated rotary brushes) also affect the efficiency of tail chalk method in estrus detection.

#### Feeding/Rumination behavior

Feeding behavior in cows is affected during estrus; therefore, it is used as a parameter to assess estrus. Specifically, feeding behavior and the number of visits to the feed and water bins were assessed, and three predictive approaches were evaluated. Feed and water intake were reduced during the estrus phase, and the number of visits to the feed bin was also reduced. It has been suggested that feeding behavior data can be considered a sole indicator for predicting estrus (36). However, Pahl et al. (37) reported variation in feeding characteristics between primiparous and multiparous cows, wherein primiparous cows fed longer time than multiparous cows; therefore, the parity of cows when assigned for estrus detection using feeding behavior should also be noted.

#### Estrus detection patches

Estrus detection patches (e.g., Estrotect) were used in beef cows, and color change was considered an indicator of estrus (38). The patches are usually colored, and the color change potentially indicates mounting by herdmates. The advantage is that the patches are weather resistant, and color changes are possible only when physically rubbed; therefore, they are efficiently used in estrus detection (39). Smith et al. (40) also used estrus detection patches to detect estrus in cows, which was further validated by serum progesterone concentrations. However, patch retention should at least be for four weeks after application to detect the estrus effectively but sometimes retention of the patches is poor owing to hair shedding in cows (41).

### Physiological and hormonal methods

#### Fern pattern

Ferning is the microscopic appearance of fern leaf-like patterns that result from the crystallization of sodium chloride in dried cervical mucus (42). If the nature of the pattern is more characteristic with intact branching of ferns associated with high estrogen, it indicates estrus, whereas, fragmented pattern of ferns indicates non-estrus. It was found in tears, nasal mucus, cervico-vaginal fluid, and saliva, and is extremely sensitive to changes in hormone levels, particularly the level of estrogen (43). Therefore, the use of fern pattern in saliva or cervical mucus has been suggested to complement estrus detection.

Salivary fern pattern has also been shown to be an indicator of estrus in crossbred Jersey (44) and zebu (45) cattle. The fern pattern in cervical mucus was also evaluated and aided in estrus detection; cows expressed typical fern patterns during estrus, whereas the fern pattern was atypical (fragmented ferns) during other stages, thereby making it a potential tool for estrus detection (46). Despite its potential for detecting or confirming estrus in individual cows, fern pattern is not a practical approach for routine estrus detection.

#### Milk progesterone analysis

Recognizing the importance of milk progesterone in the regulation of estrous cycles, a mathematical model was developed to analyze temporal fluctuations in progesterone concentrations during the estrous cycle (47). This analysis incorporated variables such as parity, age, breed, and health status to improve monitoring accuracy.

#### Monitoring real-time milk progesterone

Successively, studies have focused on the sensor-based evaluation of milk progesterone in detecting estrus. For instance, the in-line milk analysis system (IMAS) utilizes biosensor technology that detects progesterone concentration in milk samples periodically, which aids in identifying estrus in dairy cows during the spontaneous estrous cycle (48). The IMAS system (trade name: HerdNavigator™) also detected dynamic changes in milk progesterone concentration and aided estrus detection in dairy cows. This method has been scientifically validated, with progesterone levels monitored at frequent intervals, starting approximately 21 days postpartum. Recently, a near-infrared spectroscopic (NIR) system connected with the milk sampler, milk flow meter, and a computer was developed to analyze progesterone in milk samples from dairy cows, wherein the spectral data of the milk were analyzed at every 20 s interval for one minute during milking, which was used to monitor estrus status (49).

A recent review of in-line milk progesterone (IMP4) monitoring (50) characterized IMP4 as a precision tool that detects estrus and identifies return to estrus after failed insemination. Among non-pregnant cows, 85% were detected returning to estrus before 30 days post-AI (before first pregnancy diagnosis typically occurs on most dairies) enabling rapid decisions to reinseminate. IMP4 also helps in detecting the onset of cyclicity and pregnancy status, thereby support individualized reproductive management and not just simple alerting. Despite these advantages, as the technology can be used only with specific types of robotic or voluntary milking systems its application is limited to such herds only.

#### Vocalization based approaches

Physiological signs are often considered good indicators of estrus in dairy cattle. Schon et al. (51) suggested that vocalization was significantly increased starting two days before the onset of estrus but reduced after estrus, implying that it is an useful indicator of estrus. Indeed, a comparison of vocalizations during pre-estrus, estrus, and post-estrus phases revealed a higher incidence of vocalization during estrus than during non-estrus periods. Vocalization was also higher during spontaneous estrous cycles than during the induced estrous cycles (26). A collar-based cattle call monitor used the recorded vocalization calls of heifers interpreted by an observer. The sensitivity (87%), specificity (94%), and positive and negative prediction rates were 80 and 96%, respectively, and the model was proposed to be suitable for untethered cows (52). Vocalization, however, requires manpower to interpret the results, making it a time-consuming and impractical approach.

#### Infrared thermography (IRT)

Infrared thermography (IRT) is used to detect the heat emissions that are not visible to the naked eye. IRT utilizes a special camera to detect the heat and convert it into a visual image called a thermogram. It has been observed that the temperature of certain body parts in cows increases during estrus. A study that utilized muzzle and vulval temperatures to predict estrus in cows using IRT found a high sensitivity, but the specificity (29%) and positive predictive values were low (64%) in predicting estrus (53). The measurement of temperature in different body areas for nine consecutive days revealed an increased temperature in nine of eleven body areas during estrus, and the rise in temperature was associated with declining progesterone levels (90.91% sensitivity at 24 h and 84.62% sensitivity at 48 h) (54). In Holstein dairy cows, a combinatorial approach involving IRT of skin temperature (raw skin and residual skin temperature), vulva, and vulval lips and behavioral biometrics (hip and tail movements) helped detect estrus with reduced false-positive and false-negative results (55). Marquez et al. (56) also used an automated estrus detection system, in that they used IRT to measure skin temperature and tail movements defined by the authors as “Estrus BenchMark” in Holstein dairy cows. The skin temperature was elevated (*p* = 0.09) compared to baseline during pro-estrus and estrus, which, when combined with tail movement, helped detect estrus. Riaz et al. (57) highlighted the importance of IRT in detecting estrus in cows and proposed it as a non-invasive and user-friendly technique.

Vicentini et al. (58) used IRT to analyze the temperature in the muzzle, right eye, and vulva, and a digital thermometer to measure the temperature of rectum and vagina in Gyr heifers. They found the highest temperature with less environmental distortion in the eye and vulva than in other body parts and suggested IRT as a possible approach to detect estrus. However, the eye and vulva may have high blood perfusion that would have indirectly increased the temperature, and it is important to note that this study was performed in hormone-synchronized heifers. This finding warrants further evidence by validating the same idea for the spontaneous estrous cycle.

Merkelytė et al. (59) catalogued the IRT camera models used in estrus detection studies and highlighted that ambient temperature, cow behavior (lying vs. standing), and measurement angle significantly influence the thermogram readings, and that no standard correction protocol has been established across studies. Riaz et al. (57) emphasized that while IRT is non-invasive and does not depend on physical activity (making it suitable for tie-stall housing where pedometer-based systems are ineffective) the controlled capture conditions remains a barrier to on-farm application (57). An automated IRT platform (Estrus BenchMark) validated against HerdNavigator in-line milk progesterone in a robotic milking herd found that vulval skin temperature tended to increase on the day of estrus relative to seven days prior, but the increase reached statistical significance only in cows with high-confidence low progesterone estrus alerts (that is >80% quality), indicating that IRT alone may generate noise in low-quality estrus events and that confirmation remains important (55). Collectively, these findings suggest that IRT is the best deployed as a complementary rather than standalone estrus detection tool.

### Sensor based technologies

#### Wireless sensors

Several sensor devices have been developed using wireless technology, which allow for remote monitoring of estrus activity. Ultrawideband (UWB) radio technology, another radiotelemetric method that relies on mounting and standing, detected the onset of estrus in nine out of 10 cows (90% specificity) and also confirmed the cows that were not in estrus (six out of 16 cows, 37.5% specificity) (60). One of the advantages of the UWB system is that almost half of estrus behaviors occurred during the late hours and were measured effortlessly without requiring manpower. An inverted F-type antenna (which provides enhanced signals) was placed within a movement sensor and mounted to the neck belt of the cows, which transmitted movement data to help detect estrus. This type of antenna is superior to the chip antennas and is believed to enhance the signal transmission (61).

#### Walking (pedometers and collar-based activity monitors)

Pedometers are electronic devices that measure walking activity by counting the number of steps taken. The use of pedometers in detecting estrus has been appreciated for a long time. Machado et al. (62) revealed that activity monitors are helpful for detecting estrus in dairy cows after a successful synchronization program. A 2025 meta-analysis study (33) reported that activity monitor-based detection occurring within voluntary waiting periods was associated with a higher likelihood of timely artificial insemination and improved subsequent reproductive outcomes.

Considering the lameness in cows, pedometers may have a negative impact on estrus detection. In this case, lameness activity sensors [e.g., wearable motion sensors (63), accelerometers (64)] can be coupled with pedometers to detect lameness and estrus simultaneously. For instance, the use of wearable devices have been highlighted based on their wide application in dairy farms (65, 66). Indeed, estrus prediction by pedometers was inefficient in cows housed in tie stalls because cow activity did not significantly change during estrus when cows were restrained in tie stalls (6). However, it is shown that pedometers detect estrus even in silent heat cows that were group housed (5 to 6 in a group), as the steps by the cows appear to be more prominent in silent estrus in concrete-floored open house paddocks (67). Silent estrus refers to the condition in which the cow undergoes physiological changes associated with estrus, but the behavioral signs are absent or very subtle. Silent estrus can be caused by various physiological, environmental and nutritional factors (68). Roelofs et al. (69) found no effect of housing conditions in the detection of estrus in dairy cows, as the sensitivity (between 76 and 82%), specificity (between 99 and 100%), and positive predictive values (87 and 92%) remained similar for cows that were housed in two different systems (pasture or indoor).

Although accelerometers are highly useful in detecting estrus, parity, and season of insemination and other variables could affect the quality of the data acquired from the accelerometer. In addition, false positives are high, ranging from 17–55%, in pedometer-based estrus detection (70). However, activity-based monitoring of cows is the common method to detect estrus in silent heat cows, which showed reduced standing events (67).

In a survey-based study, many farmers accepted that the heat detection rate using collar-mounted sensors in cows was significantly higher than that of other methods (including visual detection and other electronic device-based methods). However, only approximately 53% of farmers confirmed the financial benefits, raising a huge concern over the profitability of farmers using these types of electronic devices for estrus detection (71). Farmers often look for economic benefits; therefore, the use of automated estrus detection tools have many advantages as if these tools may also be used to detect the health status of the herds (72).

Recent advances in collar-mounted sensors have improved the accuracy and variety of activity-based estrus detection. An evaluation of the RUMI neck-mounted accelerometer collar in high-producing Holstein cows found a specificity of 100%, sensitivity of 90.9%, and overall accuracy of 93.6% for estrus detection (73). Notably, the study also defined an optimal artificial insemination window of 11–15 h after estrus onset, providing farm managers with reproductive guidance derived directly from wearable sensor data (73). Additionally, Yu et al. (2024) (65) described a solar-powered wearable device integrating accelerometers and temperature sensors into a curved casing designed to improve signal accuracy at the neck, while reducing battery maintenance burden, one of the persistent limitations found in wearable systems in large herds.

#### Temperature sensors (ear, vulva, and rumen)

Physical parameters provide valuable information for determining estrus in cows. Despite the diurnal rhythm, the body temperature of cows usually shows subtle differences during the estrus phase. Therefore, the body temperature of the animals should be adjusted with atmospheric temperature and humidity to identify the exact temperature difference to be used in estrus detection. Intriguingly, Suthar et al. (74) found no correlation between body temperature and serum progesterone concentration. However, the vaginal temperature measured using a digital thermometer in Japanese black cows increased during estrus, and the temperature increase was constant from −1 d of estrus and remained unchanged during estrus (75). Miura et al. (76) examined ventral tail surface temperature during the estrous cycle of three different sets of cattle (Holstein Friesian heifers, Japanese Black heifers and Japanese Black cows) using a wireless sensor. When used in estrus detection, the sensitivity ranged between 56 and 89%, and the accuracy ranged from 46 to 71%. Interestingly, the residual temperature induced by circadian rhythm did not affect the tail surface temperature during estrus (increased from baseline temperature), suggesting that it is a vital tool. Notably, ear surface temperature measured using a digital surface temperature monitor has been proposed to have less potential for detecting estrus (77). A recent study suggested that increased ruminoreticular temperature (0.73 °C) measured using biocapsule sensors, in combination with body activity, aided estrus detection in Hanwoo cows (78).

An integration of temperature- and accelerometer-derived data significantly enhanced estrus detection accuracy, exceeding 90% across different breeds and production systems. This combination of thermal and activity parameters captures physiological and behavioral changes observed in estrus, ultimately reducing false detections caused by environmental fluctuations or any non-estrus related activity like chin-resting, playful mounting, or increased walking resulting from management practices (59).

From an engineering perspective, a study of bovine health monitoring technologies highlighted the rumen bolus sensors as a particularly promising avenue for internal temperature monitoring, as they are less likely to have external environmental interference compared to surface-mounted sensors on the ear or tail. However, the standardization of threshold values and correction algorithms across different breeds, housing systems, and climates remains a barrier to the widespread use of temperature-based estrus detectors (79). Furthermore, the combination of ear-sensor accelerometers (CowManager system) with in-line milk progesterone confirmation (HerdNavigator) was found to complement each other, as behavioral changes in eating, active, and highly active states were detectable in both young and mature cows during the phases of declining progesterone, although the activity pattern of dairy cattle at dawn or dusk introduced timing-related variability in sensor-based detection (80).

#### Implantable sensors

An implantable sensor is a device that is inserted into the body which can monitor physiological parameters, such as temperature, hormone levels, movement patterns, and pressure changes. For instance, the parameters, such as electrical recordings and temperature changes in an aqueous solution were measured using an implantable microsystem (81), whereas vaginal tissue resistance, temperature and acceleration were measured using a wireless intravaginal probe (9). Unlike other wireless sensors used in estrus detection regimens, the development of implantable devices is still in its infancy. It is also crucial to note that Kim et al. (78) and Andersson et al. (9) used estrus-induced cows, whereas Morais et al. (81) did not use live animals and used aqueous solutions to measure the parameters. Keeping this in mind, there are still many developments that are required for implantable sensors. In addition, it is important to assess the efficacy of these sensors in the spontaneous estrous cycle of cows.

### Image and computer vision approaches

#### Time lapse video imaging

Female–female mounting is a prominent behavioral pattern for use in image processing techniques and predicting estrus in dairy cattle. The images acquired during female–female mounting for a 2-min period can provide meaningful inferences for assessing estrus (82). Cameras were placed in the barn at specific heights and angles to cover the front, rear, and top views of the cows. A complimentary 720p metal oxide semiconductor sensor and infrared illuminator that provided illumination to 15 m at night time were equipped in the camera. Initially, the image processing was performed by observing/calculating the number of cows. Therefore, the number of cows during the initiation of mounting was physically observed as 2, and that during mounting was calculated as 1.5 (because one cow mount on another cow reduces the physical observation of two cows). By this way, watching a 2-min video would be sufficient to confirm estrus in dairy cattle. However, it has a high chance of false positives (82). Moreover, this technique is not feasible for open housing or grazing systems, where video cameras have limitations in covering a wider range of animals (83). Recently, Hanpinitsak et al. (84) also highlighted the advantages of multi-angle imaging for estrus behavior recognition, but a fully functional method is yet to be established.

Dong et al. (85) integrated an enhanced YOLOv5s model with the DeepSORT tracking algorithm achieved 93.3% average accuracy in tracking individual cow movement distances within a barn by using corner-mounted 1080p cameras. This demonstrated that multi-camera configurations can resolve the blind-spot limitations present in single-camera setups. However, there is a requirement for large, manually annotated training sets.

#### Validation of behaviors using recorded images

In some instances, captured behaviors can be assessed and validated to detect estrus as a post-screening approach. Although this approach cannot provide real-time detection, it can handle many datasets over a long period to efficiently detect estrus. Video cameras installed in the barns for a continuous period of 6 months were used to assess the estrus in cows using the video management software (Camera Icons method). The cows were considered in estrus if they stood to be mounted for more than 2 s, and a group of profile photos of each cow helped in distinguishing estrus cows from others. Therefore, image analysis helped in detecting estrus more than the visual observation method. However, the combination of both methods aided in detecting estrus at a higher rate than the individual approach (86).

Hirata et al. (87) used motion history image to extract the features of riding behavior in Japanese black cows. Riding behavior was detected using optical flow, which utilizes moving objects to assess estrus (87). An image processing system was used to detect estrus in Holstein-Friesian and Sahiwal crosses (88). The images were based on the activities of the cows, particularly during mounting. When mounting occurs, the body of the rider overlaps with that of another cow, and the overlap is calculated as 1.5 cows. Estrus was assessed based on the stipulated measured distance of the detected objects, wherein the program saved the image frame and indicated a positive estrus (88). This approach was similar to that used by Tsai and Huang (83).

Although it is prudent to note that behavioral expression varies in cows and is dependent on various factors, this technique can be used in a larger population of cows to identify estrus in real time. Wang et al. (89) used an improved method of image processing of behaviors (YOLOv5) to detect estrus. The images were extracted from mounting videos of the cows, and 675 images were taken from the training set. YOLOv5 detected estrus with high accuracy (94.3%) and precision (97%); therefore, it is considered a robust method. The advantage of this method is the faster analysis of data, which can infer 71 frames per second; therefore, it is suggested for real-time use in natural scenes to aid in efficient estrus detection.

### Biomarkers in body fluids

#### Volatile biomarkers

The physiological biomarkers are secreted into various body fluids depending on the stage of the estrous cycle. For instance, volatile compounds in body secretions, such as urine, feces, saliva, vaginal secretions, and blood, were analysed to identify biomarkers for estrus detection. All samples were collected from Holstein cows during the proestrus, estrus, and post-estrus phases and were found to have eight compounds in common. These eight compounds have been suggested as biomarkers of estrus and used to develop biosensor-based technologies for estrus detection (90). Sankar and Archunan (91) reported acetic acid, propionic acid, and 1-iodo undecane in the feces of estrus cows, which, when applied to the genitalia of dummy (non-estrus) cows, elicited flehmen and mounting behaviors in bulls.

Based on the behavioral responses of bulls, estrus-specific compounds, such as acetic acid and propionic acid, were utilized in the development of an electronic nose to aid in estrus detection (92). Ramachandran et al. (93) found oleic acid, octanoic and butanoic acids in the urine and cervical mucus of Kangayam breed cattle (Table 3). These volatile biomarkers have a high potential to detect estrus because they are present in two sources but have not yet been utilized in estrus detection regimens. In nulliparous beef heifers, cervical mucus contains three volatile organic compounds that can be used as estrus biomarkers (94).

**Table 3:** Volatile biomarkers identified in different sources of cows.

Name of the compound	Sources	Reference
Acetic acid and propionic acid	Urine, feces and cervical mucus	(91)
1-Iodo undecane	Feces	
Oleic acid, octanoic acid, and butanoic acid	Urine and cervical mucus	(42)
3-methyl pentane, hexanal, 4-methylphenol (p-cresol), phenylacetaldehyde, 3-phenylpropiononitrile, 1 H-indole, cyclotetrasiloxane octamethyl and pentane 2-methyl	Various body fluids	(90)

Swabs collected from the perineal area and cow feces were used in an e-nose system (doped with different acids) to detect estrus. The sensors showed high voltage variations when reacting with volatile compounds owing to the electric response to the compounds, whereas the voltage dropped when the samples were removed, thus providing a higher sensitivity (95). The advantage of this study is that it used independent data of compounds from other studies and evaluated their efficacy. Similarly, Ali et al. (96) used electronic sensors to detect estrus based on the odor from perineal headspace of the cows. The electronic nose (EN) MENT-EGAS prototype developed by them demonstrated that direct perineal headspace sampling with metal-oxide sensor arrays could discriminate proestrus from estrus and estrus from metestrus with high accuracy in Holstein-Friesian cows (96). A concern raised by subsequent work (97) is the influence of error sources. This includes environmental background odors, feed headspace VOCs, and animal movement, which can reduce the reproducibility of e-nose readings when used under real farm conditions rather than controlled settings (97).

#### Protein biomarkers

Lactoferrin was found to be highly expressed in the cervical mucus of estrus cows and was used in the development of monoclonal antibody-based kit for estrus detection (98). Therefore, a rapid bovine heat detection kit using the anti-bLF mAbs was developed, which was tested on the cervical mucus from 12 cows. Five to seven drops (10 μL/drop) of the mucus solution were dotted onto the circular specimen spot of the device to react with the antibody in sandwich ELISA. When cows were in estrus, the device displayed two red lines, in that the color change in the second line is dependent on the intensity of estrus. To date, this is one of the available kits based on monoclonal antibodies for the detection of estrus in cows. It has been tested in a previous study with Korean native cattle and inseminations following estrus detection using this method yielded a conception rate of 64%.

As body fluids contain many small molecules and interfering biological constituents, sensors and their components should be selected and fabricated accordingly to work with various body fluids. The sensors and their elements should also be sufficiently sensitive to work at the lowest concentration of the analyte and with a smaller sample volume. This is because it is not always possible to collect sufficient quantities of the samples. Considering the interfering substances and availability, it is better to develop a sensor for saliva, as it can be collected at any time using many non-invasive approaches. However, it is also essential to consider food-borne contaminants, irrespective of the collection time. In addition, it is not possible to collect the saliva when this approach is used in a large herd. Urine, feces, and cervical mucus contain many potential metabolites that can be used as biomarkers. It is also important to note that the physical properties of cervical mucus in cows differ between spontaneous and induced estrus, with a higher crystallization pattern in spontaneous estrus than in induced estrus (27). Above all, calibration of the sensor should take a minimum time; therefore, many samples cannot be tested under field conditions within a short time when large farms are considered.

### Machine learning approaches / Algorithms

#### BovHeat- an open-source analysis tool

The development of an open-source platform was attempted based on the activity data of cows collected by an automated activity monitoring system, wherein BovHeat (an open-source Python-based analysis tool) helped in visualizing activity data with many additional features, helping to detect estrus (99). Briefly, the activity data obtained using a neck-attached accelerometer were received in a radio frequency unit connected to a computer. Files containing the activity data were then uploaded into BovHeat, which detected the estrus cows based on the change in activity index. It included three levels of activities, such as onset, peak, and end, to evaluate the estrus. The advantage of this technique is that missing data for a particular cow can be included manually, and the threshold for the activity can be adjusted according to the farm. The BovHeat method significantly reduced the time required for interpretation compared to the manual method (99).

#### Others

Artificial intelligence (AI) and the Internet of Things (IoT) are becoming highly applicable in the agricultural sector. The introduction of machine learning approaches and algorithms has contributed to the development of estrus detection strategies. However, some data pertaining to estrus behaviors are yet to be utilized in AI platforms (100). Algorithms and approaches primarily depend on the behavioral expressions of the animals and require valid input data to appropriately interpret the captured behaviors.

Using an image processing technique and algorithm, images of the lumbar region of the cows were captured, and some of the features of the images were extracted to differentiate between different phases of the estrous cycle and detect estrus (101). It is assumed that the lumbar area of cows changes between the estrus and non-estrus phases. However, it is not considered a valid single characteristic to represent estrus. Moreover, changes in the lumbar region also occur every hour of the estrous cycle; therefore, using this imaging algorithm may not provide meaningful results in this context.

Brunassi et al. (102) utilized the selected information from the estrus cows to develop a fuzzy system, which was used to detect estrus and defined the results in three different categories; in estrus, may be in estrus, and not in estrus. When this fuzzy system was used in the field to detect estrus, it aided in the detection of 457 estrus events in 24,664 cows. The major advantage is that among the positively detected cows, 84.2% were found to be in true estrus; however, the false-negative rate was high (102).

A new algorithm was developed to detect estrus based on the creation of an effective training set that was used to develop a model to differentiate between the activities of estrus and non-estrus cows. Briefly, the activity index curve predicted estrus (two-fold increase in behaviors) (103). Higaki et al. (104) used supervised machine learning approaches and used vaginal temperature and conductivity. Three machine learning algorithms were developed, and the best model successfully detected 16 of 17 estrus events, indicating that the application of machine learning together with physiological data of the cows improves the efficiency of detecting estrus.

Thirteen features extracted from the ventral tail surface temperature data were used in the machine learning approaches, wherein the three models were unable to differentiate between estrus-dependent and estrus-independent tail surface temperatures (82). It is reasonable to believe that tail surface temperature may not be the sole indicator of estrus. In addition, the authors used two different breeds of cows (Holstein and Japanese Black), which could have provided inconclusive results. In a follow-up study, Higaki et al. (105) developed a multimodal device to measure body surface temperature and behavioral activity and used machine learning algorithms (decision tree, artificial neural network, and support vector machine) to identify estrus in tie-stalled cows. The developed algorithms showed high sensitivity when both body surface temperature and behavioral activities were combined, and that support vector machine attained higher sensitivity and precision. Together, this study proved that the combination of parameters and the use of machine learning algorithms are valid tools for detecting estrus in cows.

YOLOv5 is an object-based detection network that combine the features from the captured images to predict the estrus in dairy cows. Interestingly, YOLOv5 series-based non-contact monitoring system facilitated the detection of estrus with higher accuracy than that of other existing models (89). However, under field conditions, it is difficult to determine whether the mounting behavior was successful, which may sometimes lead to imprecise estrus detection. In addition, many farms utilize artificial insemination practices where bulls are not available for natural mounting. Although the study processed more than 2000 images to decipher the algorithm, the complex environmental conditions may also mislead the rate of estrus detection in this scenario. In another study, deep learning and augmented reality were used (YOLOv5 series) to identify mounting behavior and mounting regions of interest that detected estrus with a high accuracy (106). This shows that modern object detection models are suitable for continuous estrus detection modelling while maintaining accuracy and a good processing speed (107).

Recently, Wang et al. (108) utilized the vocalization data of cows to develop a machine learning approach to detect estrus in cows. The effectiveness of estrus detection was improved to 100% by consecutive vocalization times and the time at which maximum vocalization was expressed by the cows. In another machine-learning algorithm, pre-recorded behaviors (standing, lying down, walking, displacement, switching times between walking and standing, and standing mounts) collected from 296 cows with 325 estrus events representing 2,768 1-h time windows. These data were used in the backpropagation neural network algorithm, and estrus detection was aided by 95% accuracy, 98% sensitivity, and 95% specificity (109). Another study (110) explored an unsupervised learning approach for the same by applying anomaly detection methods. This study aimed to identify deviations from normal estrus behavior patterns without relying on predefined estrus labels. This, in turn, enhanced the specificity of estrus detection by reducing false positives, specifically in high-density and intensively managed systems, making it an attractive approach for large commercial farms.

CowXNet is another estrus detection technique that utilizes a camera attached to the pen and analyzes the recorded behaviors, but primarily uses YOLOv4 to detect cows in recorded videos and a convolutional neural network to locate the body parts of the cows. However, the sensitivity of this technique is lower than that of other existing techniques (111).

Chen et al. (5) developed a Long Short-Term Memory (LSTM) based model for estrus detection in dairy cows, which uses multi-behavior time series data including eating, activity, resting patterns to identify estrus events. By analysing behavioral patterns over time constantly rather than single spikes in activity, this model reduces false positives during real-time detection. This is particularly advantageous in farm conditions, where transient increases in activity due to management practices or environmental disturbances can stump a static machine learning model. The economic aspects of various estrus detection methods and their associated costs of investment on small, medium, and large-scale farms are presented in Table 4. The comparison of machine different learning approaches and their advantages and limitations are listed in Table 5.

**Table 4:** Comparison of the economic aspects of various estrus detection methods with forecasted investments on small, medium and large-scale farms.

Method	Equipment/Technology	Cost per element	Small scale farm (50 cows)	Medium scale farm (100 cows)	Large scale farm (1,000 cows)	Requirement of labor	Need for replacement/Repurchase	Issues with each method	Accuracy	Sensitivity	Ease of implementation
Visual observation	Video camera	100 US$ to 300 US$	Varies with no. of cows	Varies with no. of cows	Varies with no. of cows	Continuous monitoring	Lasts 5–10 years; may be replaced as needed	Observer effect; time consuming; high incidence of false positive; not applicable in open-housing or grazing system	94.3%	--	Moderate
	Computer	300 US$-500 US$	Does not vary with no. of cows	Does not vary with no. of cows	Does not vary with no. of cows	Continuous monitoring	Not frequent		--	--	
Activity based approaches	Mount detectors	3 US$-5 US$	150$- 250$	300$-500$	3,000$-5000$	Continuous monitoring	Usually, 5–7 years considering no damage. In case of any damage, it has to be replaced immediately	Depends on the expression of cow behaviors and affected by housing; depends on parity and season; inefficient in tie-stalls	--	90%	Moderate
	Tail paint	15 US$	750$	1,500$	15,000$	Continuous monitoring	Re-application needed after each time, hence has to be purchased frequently	Difficult to organize and observe in large scale farms	--	--	Low
	Radiotelemetric devices	100 US$-2000 US$ (based on specifications)	Varies based on requirement	Varies based on requirement	Varies based on requirement	Periodic monitoring	Lasts for 5–10 years and replacement can be done based on wear-and-tear	Lameness can intervene the success rate	--	--	High
	Pedometers/accelerometers	30 US$-45 US$	1,500$ - 2250$	3,000$-4500$	30,000–45,000$	Continuous monitoring	Every 5 years	Depends on the expression of cow behaviors and affected by housing; depends on parity and season; inefficient in tie-stalls	87–92%	76–82%	Moderate
Experimental approaches	Microphone for detecting vocalization	20 US$	1,000$	2,000$	20,000$	Continuous monitoring	Microphones last for 5–10 years, and replacement can be done according to wear-and-tear	Differentiation between social calls and estrus calls; differentiation of calls in group housed animals is difficult	80%	87%	Low
	Software to process vocalization	130 US$	Does not vary with no. of cows	Does not vary with no. of cows	Does not vary with no. of cows		Rare; Software only requires updation		--	--	Moderate - High
	Temperature sensors	50 US$-150 US$	2,500$-7500$	5,000$-15000$	50,000$-150000$	Periodic monitoring	2–3 years	Increased false positive	46–71%	56–89%	High
	Infrared thermography	50 US$-200 US$	Varies based on requirement	Varies based on requirement	Varies based on requirement	Periodic monitoring	Lasts for 10–15 years and replacement can be done in case of damage	Increased false positive	--	--	High
	Implantable sensors	200 US$-1000 US$	10,000$- 50000$	20,000$-100000$	200,000$-1000000$	Periodic monitoring	Lasts up to several years in case of no infection. Needs replacement in case of any infection	Invasive, require surgery, not feasible in large farms	--	--	Low-moderate
	Machine learning algorithms	500 US$-10000 US$	Does not vary with no. of cows	Does not vary with no. of cows	Does not vary with no. of cows	Periodic monitoring	Very rare	Require large data set to be used in the training; relies on the behavioral or physiological indicators	--	--	Moderate - high

**Table 5:** Comparison of different machine learning (ML) approaches, their advantages and limitations.

ML tool	Reference	Use of estrus sign	Sensitivity	Advantages	Limitations
BovHEAT^#^	(99)	Activity index from accelerometer	Not available	The threshold for the activity can be adjusted according to the farm	Requires prior sensor data collection, dependent on data quality
Lumbar image processing	(101)	Morphological changes in lumbar region of cows	Not available	Non-invasive method to detect estrus	Detection based on only lumbar changes is not reliable and changes in lumbar region occur every hour in estrus
Fuzzy logic system	(102)	Combined behavioral data classification	Not available	Observed high true positives (84.2% true estrus)	High false negatives
Activity index algorithm	(103)	Differentiation between estrus and non-estrus behavior	Not available	Easy to implement	Study uses 10 cows and reports 0 false positive; further validation needed to understand the scaling capacity of the method
ML with vaginal temperature and conductivity	(104)	Physiological data	~94.1%	Successful detection of true estrus (16 out of 17 estrus detected); combination of ML + physiological cues give efficient detection	Requires invasive or semi-invasive measurement
ML with tail surface temperature	(82)	Changes in tail surface temperature during estrus	Not available	Non-invasive measurement	Unreliable as a sole parameter
Multimodal ML	(104)	Change in temperature and physiological changes	Not available	Detection with high sensitivity when combined body surface temperature and behavioral activities	Requires multiple sensors; increased complexity
YOLOv5^^^	(89)	Mounting behavior	Not available	Detection with high accuracy	Difficult to determine through imaging if mounting was successful; not suitable for complex environments
YOLOv8^	(107)	Mounting behavior	Not available	Improved accuracy;high processing speed	Requires high computation; still unsuitable for complex environments
ML based on vocalization	(108)	Vocalization pattern in estrus	~100%	Achieved very high accuracy;non-invasive method	Individual animal identification is complex; sound isolation is needed without any external disturbance or noise
ML based on behavioral data	(109, 110)	Behavioral data (walking, standing, etc)	~ 98%	High accuracy, specificity and sensitivity;reduced false positives	Large, labelled datasets needed
CowXNext*	(111)	Visual behavior and body part tracking	Not available	Automated video analysis	Lower sensitivity compared to other models
LSTM-based model	(112)	Time-series behavior (Feeding, resting, etc.,)	Not available	Fewer false positives in real-time usage;advantageous in farms where static machine learning model can be affected due to environmental disturbances or increase in activity occurs due to management practices	Requires continuous datasets and has a complex training model

^#^ SCR Engineers Ltd., Netanya, Israel.

^^^ Ultralytics Inc., London, UK.

*Thanawat Lodkaew, Kitsuchart Pasupa and Chu Kiong Loo, Malaysia and Thailand.

Other listed approaches are existing algorithms that have been modified for estrus detection.

### Implementation challenges

A comparative assessment of the AI-based methods reviewed here also reveals the differences across data quality requirements, sensor costs, data labelling demands, and its practical implementation challenges. Computer vision and deep learning approaches (e.g., YOLOv5, YOLOv8, CowXNet and LSTM-based models) require large, high-quality annotated image or video datasets, often consisting of thousands of frames in order to train models. The labelling of this data is both time-consuming and requires annotators that must correctly identify mounting events, body part positions, or behavioral transitions, which in turn requires standardization. In contrast, the activity-based machine learning models that rely on accelerometer or pedometer data are easier to label since activity peaks can be verified with known estrus events that are confirmed by progesterone measurements. However, the temporal resolution and data continuity can critically affect model performance, and any section of recordings that are missing or noise, reduce the prediction accuracy. Milk progesterone-based systems such as HerdNavigator produce biochemically precise and reproducible data but are dependent on in-line biosensors integrated with the milking system, making them expensive to acquire. Similarly, wearable sensor systems such as collar-mounted accelerometers or tail surface temperature sensors, incur moderate to high upfront hardware costs and require calibration, battery replacement, and veterinary expertise (in case of implantable sensors) periodically. Signal interference and sensor displacement due to animal movement or grooming as well as inconsistent attachment in large free-stall herds also remains an implementation challenge. Vocalization-based approaches require specialized acoustic sensors capable of filtering farm noise, and their sensitivity is limited in open or noisy housing environments. Although infrared thermography is non-invasive, it requires thermal imaging cameras, controlled conditions, and careful correction for diurnal and environmental temperature changes, limiting its practical deployment.

Across all AI-driven methods, an important challenge is that most published model systems have been validated on limited breeds, housing systems, or geographic settings, and performance tends to decline when transferred to different farm conditions without retraining. Collectively, while AI and ML methods offer substantial improvements in sensitivity and reduced labor dependency, their successful implementation depends on farm-specific data, durable sensor hardware that is also affordable, and precise annotations.

#### Limitations of traditional methods

When we consider traditional methods of detection, the conventional approaches, primarily rely on visual observation, manual examination, and simple aids that detect mounting activity. These include visual monitoring of behavioral signs, the use of teaser bulls or androgenized cows, rectal palpation, vaginal mucus evaluation, tail paint/chalk, mount detectors, and vaginal electrical resistance measurements. While these methods have been widely used due to their low cost, minimal technological requirements, and ease of adoption in small-scale farming systems, they are often labor-intensive, subjective, and less reliable under conditions such as silent estrus or large herd sizes. In addition, many of these methods are not scalable to large herds and may pose safety or welfare concerns (for instance, the use of bulls and invasive examinations). These limitations have driven the development of automated and sensor-based technologies. Nevertheless, they form the foundation of estrus detection strategies and are still used either independently or in combination with modern technologies.

#### Advantages of AI and ML tools over traditional estrus detection methods

AI and ML based estrus detection methods offer significant advantages over traditional methods such as visual observation and sensor systems based on single parameter. ML-based models utilize behavioral, physiological data enabling continuous and real-time monitoring and help improve detecting estrus event efficiently as compared to conventional methods (104, 109). These systems can identify subtle and complex patterns related to estrus that cannot be detected easily through manual observation, reducing a chance for human error and improving efficiency of detection (105). Furthermore, advanced approaches such as multimodal learning, time-series models enhance robustness under diverse farm conditions, making AI-driven methods a more reliable solution for estrus detection in modern dairy farms (108, 112).

## Conclusion

Conventional approaches for estrus detection, such as visual observation, tail paint, and mount detection patches offer simplicity but are labor intensive with high risk of false positives, especially in large herds. Advanced tools, including collar accelerometers, milk progesterone sensors, and infrared thermography, improve sensitivity for silent estrus, with activity monitors linking to better timing and fertility in meta-analyses. Experimental innovations such as volatile biomarkers, fern patterns, vocalization monitors, and machine learning approaches (e.g., YOLOv5 and LSTM) show promise for non-invasive, real-time detection, though validation in spontaneous estrous cycles is needed. The same goes for AI-based approaches, which are less labor intensive but need thorough training and validation. Factors like parity, housing, lameness, and environment, reduce behavioral expression, leading to detection rates below 50% visually and false positives up to 55% in some traditional methods. With the advent of better trained models for detection, we see an increase in accurate detection and reporting of estrus in large farms is getting easier.
